# Clinico-radiological Prognostic Indicators of Surgical Outcomes in Cervical Spondylotic Myelopathy

**DOI:** 10.7759/cureus.97480

**Published:** 2025-11-22

**Authors:** Tanveer Singh, Sourabh Guria, Narendra Kumar Das, Deepak K Das, Hare R Singh

**Affiliations:** 1 Neurosurgery, Kalinga Institute of Medical Sciences, Bhubaneswar, IND

**Keywords:** cervical spondylotic myelopathy, effective canal diameter, nurick’s grading, prognostic indicators, prognostic scoring system, t2 mri signal changes

## Abstract

Background

Cervical spondylotic myelopathy (CSM), a degenerative condition of the cervical spine, has the potential to cause catastrophic and irreversible neurological impairment. In this study, an attempt was made to assess the surgical outcomes in CSM patients and evaluate the associated clinical and radiological prognostic factors impacting the outcome.

Methodology

This study was conducted between September 2022 and August 2024 in the Department of Neurosurgery of a tertiary-level hospital in Bhubaneswar among admitted patients aged 18 to 70 years with CSM. The functional neurological status was assessed using the Madras Institute of Neurology Prognostic Scale for cervical spondylotic myelopathy and pre- and postoperative outcome assessment by Nurick’s Grading system. Data were compiled and analyzed using SPSS software version 21.0 (IBM Corp., Armonk, NY, USA). Descriptive data are represented in mean ± SD, frequencies, and percentages. The chi-square test was used for association. A p-value less than 0.05 was considered statistically significant.

Results

Of the 120 enrolled patients, around 76 (63.3%) were males. Outcome assessment using Nurick’s grade postoperatively reported a significantly (p = 0.040) improved outcome in 53 (44.1%) patients. The improved outcome was seen more in patients with Nurick’s grade 3 (28/54, 51.8%). Age less than 40 years old (p = 0.014), shorter duration of symptoms (<12 months) (p = 0.037), one level of compression (p = 0.001), and the absence of T2 intramedullary hyperintense changes (p < 0.001) significantly affected the post-surgical outcome.

Conclusions

A post-surgical improved outcome was seen in more than two-fifths of the patients. Age less than 40 years old, shorter duration of symptoms (<12 months), one level of compression, and the absence of T2 intramedullary hyperintense changes were the factors that significantly affected the post-surgical outcome of the patients. These clinical and radiological factors can guide the treating doctor to judge the potential of the patients and plan appropriate interventions.

## Introduction

A degenerative condition of the cervical spine, cervical spondylotic myelopathy (CSM) has the potential to cause catastrophic and irreversible neurological function impairment. CSM is the primary cause of acquired spinal cord dysfunction and a significant cause of disability in the adult population [1]. Nearly everyone aged over 40 years, sooner or later, has cervical spine problems. When cervical spondylosis develops, a patient who already has congenital or acquired cervical canal narrowing is at risk for neurological impairment [2,3]. Cervical canal narrowing from disc protrusion, facet joint, ossification of posterior longitudinal ligaments, or degenerative spondylosis is believed to cause CSM [4].

Before seeking medical help, myelopathy patients usually exhibit symptoms and indicators for several years. Even though the disease usually progresses slowly, if treatment is not received, the course frequently involves a progressive decline. A tiny proportion of patients show signs and symptoms that progress more quickly. Many surgical techniques have been developed to expand the cervical spinal canal anteriorly or posteriorly because most patients who present with the signs and symptoms of CSM have some degree of permanent disability, and conservative treatment is unlikely to resolve symptoms [5].

Although preoperative severity is directly linked to the surgical outcome, little is known about the role of other factors that affect the outcome. Numerous investigations have been conducted to identify the prognostic factors of the outcomes [6]. Numerous factors, including the patient’s age, the length of time they have been experiencing symptoms, spinal cord pathological changes, the cervical axial canal area, the anteroposterior diameter, intramedullary high signal intensity on T2-weighted T2 MRI, and their impact on prognosis, have been studied before by various studies [7]. In this study, an attempt was made to assess the surgical outcomes in CSM patients and evaluate the associated clinical and radiological prognostic factors impacting the outcome.

## Materials and methods

Study setting and duration

A prospective, observational study was conducted between September 2022 and August 2024 in the Department of Neurosurgery of a tertiary-level hospital in Bhubaneswar.

Study population

The study population consisted of patients with CSM who were admitted for surgery to the tertiary hospital between September 1, 2022, and August 31, 2024. All eligible patients were included following the universal sampling method.

Inclusion and exclusion criteria

The study included all patients with CSM, aged 18 to 70 years, admitted to the tertiary-level hospital in the Department of Neurosurgery from September 2022 to August 2024. We excluded any recurrent case of CSM, patients with traumatic injury to the cervical spine, patients with spinal tumors, Pott’s spine, multiple-level prolapsed intervertebral discs (cervical, dorsal, lumbar), and craniovertebral junction abnormalities.

Study tool

The functional neurological status was assessed using six prognostic factors, adapted from the Madras Institute of Neurology Prognostic Scale for CSM (MINPS). With the current clinical and radiological data, this scale is highly applicable and has a good prediction accuracy for results [8]. The prognostic factors have been outlined in Table 1.

**Table 1:** The Madras Institute of Neurology Prognostic Scale for cervical spondylotic myelopathy (MINPS).

Prognostic factors	Subdivisions
Age	<40 years
	41–60 years
	>60 years
Sex	Male
	Female
Duration of symptoms	<12 months
	12 months to 2 years
	>2 years
Nurick’s grade	0–2
	3
	4–5
Bladder/Bowel symptoms or both	Yes
	No
Effective canal diameter	<9 mm
	9–11 mm
	>11 mm
Surgical approach	Anterior
	Posterior
Number of levels of compression	1
	2
	3 or more
Intramedullary signal changes	No change
	T2 signal ill-defined lesion (type 1)
	T2 signal well-defined lesion (type 2)

Nurick’s grading system was used to evaluate the severity of cervical myelopathy preoperatively and postoperatively. This system was first described by Nurick in 1972 to categorize myelopathy patients, primarily by the level of disability and walking difficulty, giving medical professionals a methodical approach to determining the severity of a disease [9]. The grading system is outlined in Table 2.

**Table 2:** Nurick’s disability score.

Grade	Signs and symptoms
0	Signs or symptoms of root involvement, but no evidence of spinal cord disease
1	Signs of spinal cord disease, but no difficulty walking
2	Slight difficulty in walking that prevented full-time employment
3	Difficulty walking prevented full-time employment or the ability to do all housework, but it was not so severe that it required someone else’s help walking
4	Able to walk only with someone else’s help or with the aid of a frame
5	Chairbound or bedridden

Study outcome

The outcome was categorized into the following three groups using Nurick’s grading pre- and postoperatively: (1) improvement (at least +1 of the grade), (2) stationary (0), (3) deterioration (at least -1 of the grade). The outcome was recorded as per the latest postoperative follow-up.

Study protocol

**Figure 1:**
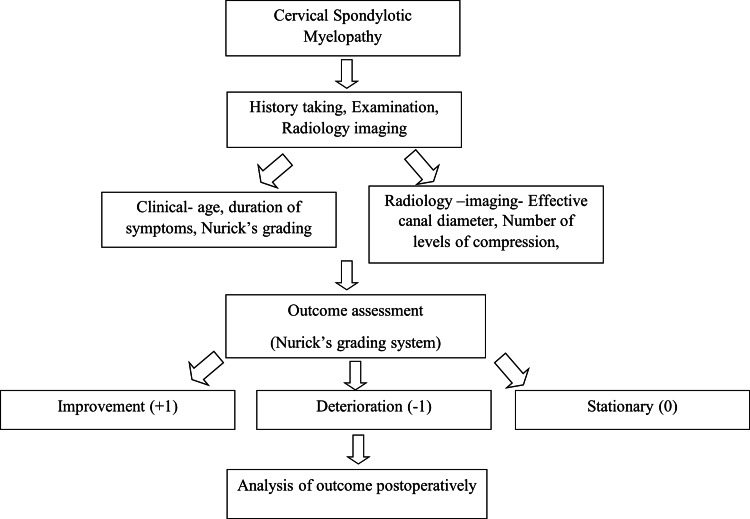
Study protocol.

The study protocol is outlined in Figure 1.

Statistical analysis

Data were compiled into Microsoft Excel (Microsoft Corp., Redmond, WA, USA) and analyzed using the SPSS software version 21.0 (IBM Corp., Armonk, NY, USA). Descriptive data were represented as mean ± SD, frequencies, and percentages. The chi-square test was used to test the association. A p-value less than 0.05 was considered statistically significant.

Ethical considerations

Institutional Ethical Clearance was obtained from Kalinga Institute of Medical Sciences (approval number: KIIT/KIMS/IEC/1062/2022) before commencing the study, which was conducted according to the Declaration of Helsinki guidelines. Informed consent was obtained from all participants, and confidentiality was strictly maintained throughout the study.

## Results

Of the 120 eligible patients with diagnosed CSM, around 76 (63.3 %) were males, and 44 (36.7%) were females. The mean age of the study population was 48.1 ± 14.4 years (range = 18-70). Of all patients, around 49 (40.8%) were less than 40 years old. The mean duration of presentation of symptoms was 1.1 ± 0.3 months. The patients were followed up for a mean duration of 10 ± 2 months. Nurick’s grade was assessed preoperatively in the study cohort. The majority of the participants had grade 4 (45%), followed by grade 3 (30.8%) and grade 5 (24.2%). The mean effective canal diameter for the study cohort was 9.7 ± 1.43 mm. The mean number of compression levels was 2 ± 0.9. The details of the prognostic factors are outlined in Table 3.

**Table 3:** Distribution of study participants according to the prognostic factors (n = 120).

Prognostic factors	Subdivisions	Frequency (%)
Age	<40 years	49 (40.8)
	41–60 years	40 (33.4)
	>60 years	31 (25.8)
Sex	Male	76 (63.3)
	Female	44 (36.7)
Duration of symptoms	<12 months	112 (93.4)
	12 months to 2 years	07 (5.8)
	>2 years	01 (0.8)
Nurick’s grade	0–2	37 (30.8)
	3	54 (45)
	4–5	29 (24.2)
Bladder/Bowel symptoms or both	Bowel only	33 (27.5)
	Bladder only	42 (35.0)
	Both bowel and bladder	45 (37.5)
Effective canal diameter	<9 mm	50 (41.7)
	9–11 mm	53 (44.2)
	>11 mm	17 (14.1)
Surgical approach	Anterior	73 (60.8)
	Posterior	47 (39.2)
Number of levels of compression	1	51 (42.5)
	2	22 (18.3)
	3 or more	47 (39.2)
Intramedullary hyperintense signal changes in T2 MRI	No change	62 (51.6)
	T2 signal ill-defined lesion (type 1)	29 (24.2)
	T2 signal Well-defined lesion (type 2)	29 (24.2)

**Figure 2:**
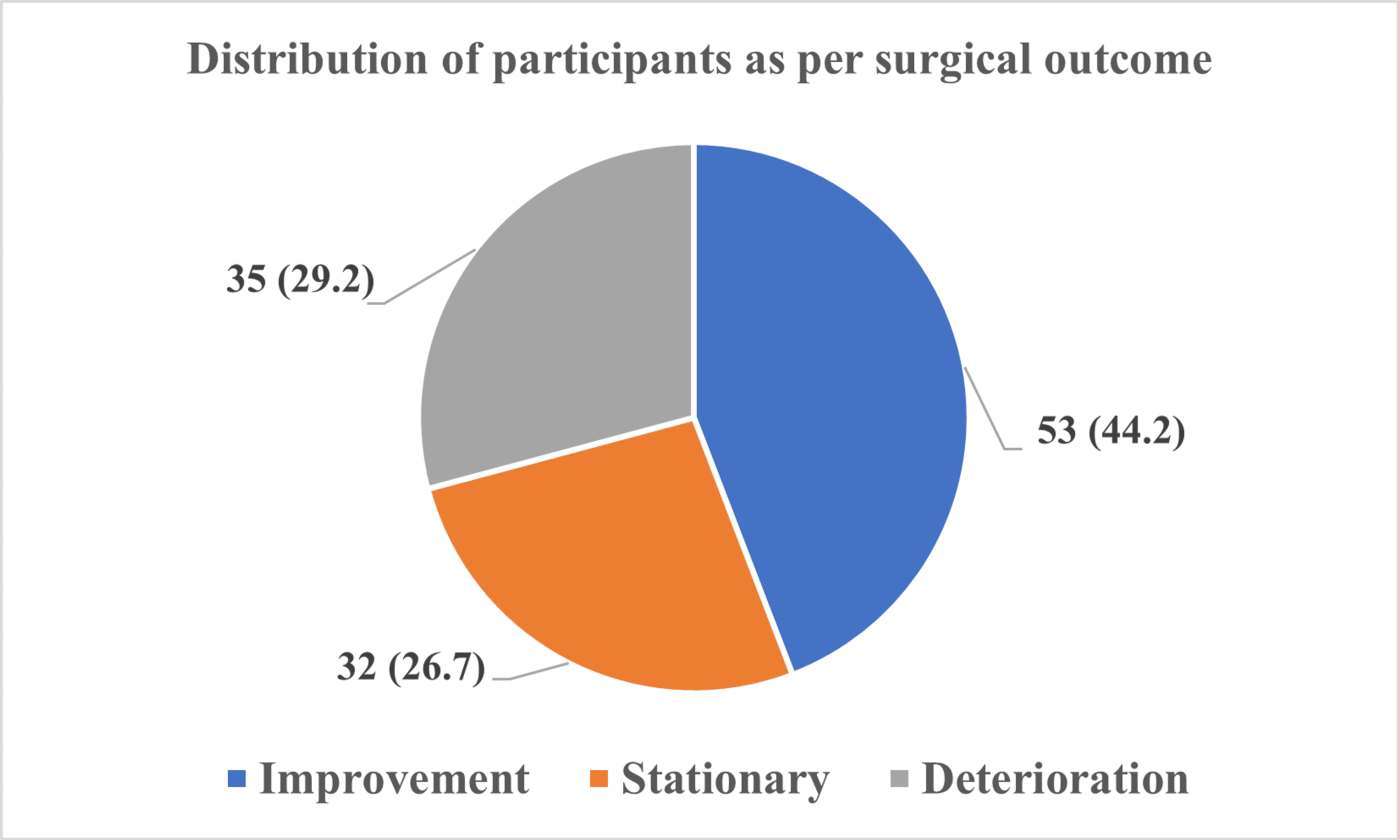
Distribution of study participants according to the surgical outcome.

Following the surgical procedure, the clinical outcome was assessed as improved, with no change (stationary) or deterioration, as depicted in Figure 2.

On assessing the outcome using Nurick’s grade postoperatively, the outcome improved in 53 (44.1%) patients, deteriorated in 35 (29.2%) patients, and remained stationary in 32 (26.7%) patients. The association was also found to be statistically significant (p = 0.040). The improved outcome was seen more in patients with Nurick’s grade 3 (28/54, 51.8%) compared to other grades.

On testing the association between outcome and preoperative/prognostic factors, the outcome was significantly associated with age group (p = 0.014), duration of symptoms (p = 0.037), number of compression levels (p = 0.001), and MRI changes (p < 0.001), as outlined in Table 4.

**Table 4:** Association between prognostic/preoperative factors and outcome. Association was tested using the chi-square test. P-values <0.05 are considered statistically significant.

Prognostic factors	Subdivisions	Outcome			χ^2^ (p-value)
		Deterioration (n = 35), N (%)	Stationary (n = 32), N (%)	Improved (n = 53), N (%)	
Age	<40 years	7 ( 20.00)	13 (40.62)	29 (54.72)	12.482 (0.014)
	41–60 years	16 (45.71)	13 (40.62)	11 (20.75)	
	>60 years	12 (34.29)	06 (18.76)	13 (24.53)	
Sex	Male	20 (57.14)	21 (65.62)	35 (66.04)	0.816 (0.665)
	Female	15 (42.86)	11 (34.38)	18 (33.96)	
Duration of symptoms	<12 months	31 (88.57)	30 (93.76)	51 (96.23)	2.916 (0.037)
	12 months to 2 years	04 (11.43)	01 (03.12)	02 (03.77)	
	>2 years	00 (00)	01 (03.12)	00 (00)	
Nurick’s grade	0–2	13 (37.14)	12 (37.50)	12 (22.64)	3.420 (0.040)
	3	14 (40.00)	12 (37.50)	28 (52.83)	
	4–5	08 (22.86)	08 (25.00)	13 (24.53)	
Bladder/Bowel symptoms or both	Bowel only	08 (22.86)	11 (34.37)	14 (26.42)	2.052 (0.726)
	Bladder only	12 (34.28)	09 (28.12)	21 (39.62)	
	Both bowel and bladder	15 (42.86)	12 (37.51)	18 (33.96)	
Effective canal diameter	<9 mm	19 (54.28)	13 (40.62)	18 (33.96)	3.759 (0.440)
	9–11 mm	12 (34.28)	15 (46.87)	26 (49.05)	
	>11 mm	04 (11.44)	04 (12.51)	09 (16.99)	
Surgical approach	Anterior	13 (37.14)	12 (37.50)	22 (41.51)	0.219 (0.896)
	Posterior	22 (62.86)	20 (62.50)	31 (58.49)	
Number of levels of compression	1	08 (22.85)	10 (31.25)	33 (62.26)	18.770 (0.001)
	2	10 (28.57)	04 (12.50)	08 (15.09)	
	3 or more	17 (48.58)	18 (56.25)	12 (22.65)	
Intramedullary hyperintense signal changes in T2 MRI	No change	10 (28.57)	12 (37.50)	40 (75.47)	25.918 (<0.001)
	T2 signal ill-defined lesion (type 1)	15 (42.85)	07 (21.87)	07 (13.21)	
	T2 signal well-defined lesion (type 2)	10 (28.58)	13 (40.63)	06 (11.32)	

## Discussion

The management of CSM remains controversial as clinicians are still debating the superiority of non-operative versus operative measures. In the case of operative procedures, there is still no consensus about using an anterior or posterior approach. The use of CT and MRI for spinal disorders has enabled the early diagnosis of age-related changes in the spine and defined new operative criteria for the surgical decision-making process. A variety of factors that include the patient’s age, symptom duration, preoperative Nurick’s grading, effective canal diameter, number of levels of compressions, bowel and bladder involvement, type of surgical approach used, and preoperative high signal intensity within the spinal cord in MRI have been shown to affect the outcome of surgical treatment of CSM.

Assessing the severity of cervical myelopathy using Nurick’s grade postoperatively, it was found that the outcome improved in 53 (44.1%) patients, deteriorated in 35 (29.2%) patients, and remained stationary in 32 (26.7%) patients. The association between outcome and severity of cervical myelopathy was also found to be statistically significant (p = 0.040). The improved outcome was seen more in patients with Nurick’s grade 3 (28/54, 51.8%) compared to other grades. A study by Ramesh et al., however, reported a significantly (p < 0.05) higher improvement among patients with Nurick’s grade 0-2 (19/34, 55.8%), followed by those with grade 3 severity (14/38, 36.8%) [8]. Variations in patient selection and the severity of the disease at presentation could be the reasons for the discrepancy between our findings and those of Ramesh et al. Patients with moderate myelopathy (Nurick’s grade 3) in the study showed a higher chance of recovery after prompt surgical decompression. It is also possible that variations in follow-up periods, rehabilitation regimens, and surgical techniques affected postoperative results.

The mean age of the study population was 48.1 ± 14.4 years in the present study. The majority of the study population (40.8%) belonged to the 40-year age group. Improvement after surgery was seen most commonly in younger patients, and this improvement was statistically significant compared to the elderly patients (p = 0.014). Middle-aged and older people are susceptible to CSM. Age is a significant predictive factor in determining how CSM surgery will proceed [9,10]. A study by Ahn et al. also reported better results post-CSM surgery in patients under 40 years of age [10]. Hasegawa et al. [11] and Holly et al. [12] reported that older CSM patients have a higher frequency of neurological problems but no discernible difference in surgical results from younger CSM patients. This could be attributed to two factors: (1) age-related changes in the spinal cord of the elderly may include a decrease in C-motor neurons, a decrease in anterior horn cells, and a decrease in myelinated fibers in the corticospinal tract and posterior cord; and (2) an increased likelihood of unrelated comorbidities in older patients that may have an impact on quality of life [13,14].

In this study, most patients had a <12-month duration of symptoms before presenting to the hospital. Compared to those who had longer presentations of CSM, patients with a shorter disease duration showed more improvement after surgical treatment. Similar findings have been reported by many other studies [15-17]. Although it did not appear to impact the surgical outcome, Karpova et al. [18] suggested that the length of symptoms was linked favorably with preoperative functional level. This might be because prolonged compression causes permanent alterations in the spinal cord. A series of pathological alterations, such as persistent mechanical deformation, inflammatory edema, and gradual microvascular impairment that results in spinal cord ischemia, are known to be triggered by chronic cord compression. Therefore, the prognosis is better for those who present with shorter symptom duration.

In this study, all patients had a preoperative Nurick’s grade of 3 or above, indicating severe CSM. Patients with preoperative Nurick’s grades of 4 and 5 showed more improvement after surgery compared to those with grade 3 CSM. This observation may be attributed to the larger functional deficit present at baseline in higher-grade patients, which provides greater potential for measurable postoperative recovery. Moreover, advanced CSM is often associated with substantial but reversible pathophysiological changes, such as significant neural compression, inflammatory edema, and compromised spinal cord perfusion, that respond favorably once decompression is achieved. In contrast, patients with grade 3 myelopathy begin with relatively better neurological function, leaving a narrower margin for observable improvement after surgery. Holly et al. also reported that patients who had a lower Nurick’s grade also had better results [12]. There does not seem to be a single baseline score index that is thought to be infallible, despite the majority of studies reporting that worse outcomes are linked to more severe starting scores.

The post-surgical improvement was most noted in patients who had an effective canal diameter between 9 and 11 mm, followed by those with a diameter <9 mm. It has been discovered that effective cervical canal diameter plays a critical role in dictating the prognosis following CSM surgery [9]. A canal diameter of less than 10 mm is deemed noteworthy and linked to a range of neurological impairments. The effective canal diameter is calculated from the prominence of the posterior osteophyte to the closest point on the spine-laminar line. Effective canal diameter is one of the key prognostic markers, and a better prognosis is noted when the effective canal diameter is more than 11 mm, as reported by Ahn et al. [10]. However, a study by Zileli et al. suggested that there is no proof linking the anteroposterior diameter of the spinal canal to neurological consequences, despite the expectation that a decrease in canal diameter will result in myelopathy [19].

Compression against the C5-6 and C6-7 discs is typically the cause of CSM. This is because, during neck flexion and extension, osteophyte production occurs more quickly at the point of maximal movement [8]. Around 42.5% of the study participants had one compression level. The treatment outcome was significantly better (p = 0.001) in patients with one level of compression compared to those who had two or more compressions. According to Fujiwara et al. [20] and Ahn et al. [10], patients who had one or two levels of cord compression fared better than those who experienced three or more levels of compression.

Spinal cord affection is indicated by high signal intensity on T2-weighted MRI and low signal intensity on T1-weighted MRI, with the T2 hyperintensity typically reflecting increased water content due to edema, inflammation, or myelomalacia from chronic cord compression. In this study, around 51.7% of the participants had an ill-defined T2 signal. Post-surgical improvements were statistically significantly higher (p < 0.001) among patients with ill-defined T2 hyperintensity compared to those with well-defined or absent T2 signal changes. The association between a high signal intensity on T2-weighted MRI images and the result of the surgery is a topic of disagreement among studies [21,22]. In the present study, most patients had ill-defined T2 signals. In a previously reported study, Kohno et al. [22] noted that intervertebral disc herniation and a brief illness duration are linked to reversible changes and that the reversibility of a condition cannot be predicted using MRI signal changes alone. The association between the signal alterations on the preoperative T2-weighted images and the clinical outcome was also shown to be ambiguous by Morio et al. [23] and Yone et al. [24].

In this study, the anterior approach was the most common surgical approach used. However, there was no statistically significant difference (p = 0.896) in the postoperative improvement between the two approaches. When CSM only includes one or two levels, the anterior approach and anterior compression are typically used. However, the anterior approach is linked to higher rates and unpredictable complications, and issues occur when more than two levels are involved. This is because of the longer graft and plate structures needed for multilevel anterior surgeries, which are more vulnerable to implant dislodgement, subsidence, and pseudarthrosis. Furthermore, changed biomechanics lead to neighboring segment degeneration, and prolonged soft-tissue retraction raises the risk of dysphagia and recurrent laryngeal nerve palsy. Compared to one- or two-level treatments, multilevel anterior surgery is more complicated and prone to complications [25]. According to the study by Shamji et al. [26], treating kyphotic patients with an anterior or combination approach produced better outcomes. There were no appreciable variations in the postoperative neurological clinical status between the anterior and posterior procedures in multilevel (more than two levels) CSM, according to a meta-analysis analyzing 10 trials [27].

Further robust studies are warranted in a larger population to validate the MNIPS, with the prognostic factors for surgical outcome in CSM patients, as well as outcome assessment by Nurick’s grading system. This will help in planning additional rehabilitation and determining the potential outcome following surgery. Moreover, these prognostic factors are clinically valuable because they help identify patients who are most likely to benefit from timely surgical intervention. Factors such as shorter symptom duration, fewer levels of compression, and the presence of reversible T2 signal changes represent conditions where early diagnosis and prompt decompression can prevent progression to irreversible spinal cord injury. Recognizing high-risk or modifiable features, such as prolonged symptoms or multilevel disease, also enables clinicians to optimize perioperative planning, institute early rehabilitation, and provide closer neurological monitoring. By addressing correctable elements early and tailoring management strategies accordingly, the overall surgical outcome for CSM patients may be improved.

A major strength of this study is its prospective design with standardized clinical and radiological assessment of prognostic factors in CSM patients. Additionally, the use of validated grading systems (MINPS and Nurick’s) enhances the reliability and comparability of the outcome evaluation.

Limitations

No long-term follow-up was done on patients to assess the implications of the surgical treatment. The severity of CSM was assessed only using Nurick’s grading for a simpler and faster interpretation of results. The accuracy and reliability of severity assessment and postoperative outcome evaluation may be restricted by their subjective nature and susceptibility to interobserver variation. Another drawback of this study is that it only used univariate (chi-square) analysis to find variables related to surgical outcome. Because of the small sample size and categorical data distribution, multivariate regression analysis was not possible. Multivariate analysis should be used in future research with larger sample sizes to assess prognostic factors more thoroughly.

## Conclusions

A post-surgical improved outcome was seen in more than two-fifths of the patients. Age less than 40 years old, shorter duration of symptoms (<12 months), one level of compression, and absence of T2 intramedullary hyperintense changes were the factors that significantly affected the post-surgical outcome of the patients. Factors such as the sex of the patient and the type of surgical approach had no significant impact on the postoperative outcome of the patients. These clinical and radiological factors can guide the treating doctor to assess patients and plan appropriate interventions. For instance, patients presenting early with a single-level compression and no T2 hyperintensity can be prioritized for timely surgical decompression, as they are more likely to experience meaningful neurological recovery. In contrast, patients with prolonged symptom duration, multilevel disease, or T2 signal changes may require more detailed preoperative counseling, individualized surgical planning, and early initiation of structured postoperative rehabilitation to optimize outcomes. Understanding these prognostic markers can enable clinicians to tailor management strategies and improve overall post-surgical results in CSM patients.

## References

[1] McCormick JR, Sama AJ, Schiller NC, Butler AJ, Donnally CJ 3rd (2020). Cervical spondylotic myelopathy: a guide to diagnosis and management. J Am Board Fam Med.

[2] Hoy DG, Protani M, De R, Buchbinder R (2010). The epidemiology of neck pain. Best Pract Res Clin Rheumatol.

[3] Lu X, Tian Y, Wang SJ, Zhai JL, Zhuang QY, Cai SY, Qian J (2017). Relationship between the small cervical vertebral body and the morbidity of cervical spondylosis. Medicine (Baltimore).

[4] Mattei TA, Goulart CR, Milano JB, Dutra LP, Fasset DR (2011). Cervical spondylotic myelopathy: pathophysiology, diagnosis, and surgical techniques. ISRN Neurol.

[5] Tu J, Vargas Castillo J, Das A, Diwan AD (2021). Degenerative cervical myelopathy: insights into its pathobiology and molecular mechanisms. J Clin Med.

[6] Zika J, Alexiou GA, Giannopoulos S, Kastanioudakis I, Kyritsis AP, Voulgaris S (2020). Outcome factors in surgically treated patients for cervical spondylotic myelopathy. J Spinal Cord Med.

[7] Sun Q, Hu H, Zhang Y, Li Y, Chen L, Chen H, Yuan W (2011). Do intramedullary spinal cord changes in signal intensity on MRI affect surgical opportunity and approach for cervical myelopathy due to ossification of the posterior longitudinal ligament?. Eur Spine J.

[8] Ramesh VG, Kannan MG, Sriram K, Balasubramanian C (2017). Prognostication in cervical spondylotic myelopathy: proposal for a new simple practical scoring system. Asian J Neurosurg.

[9] Kun YT (2005). Analysis of 13 prognostic factors in cervical spondylotic myelopathy. J Korean Soc Spine.

[10] Ahn JS, Lee JK, Kim BK (2010). Prognostic factors that affect the surgical outcome of the laminoplasty in cervical spondylotic myelopathy. Clin Orthop Surg.

[11] Hasegawa K, Homma T, Chiba Y, Hirano T, Watanabe K, Yamazaki A (2002). Effects of surgical treatment for cervical spondylotic myelopathy in patients > or = 70 years of age: a retrospective comparative study. J Spinal Disord Tech.

[12] Holly LT, Matz PG, Anderson PA (2009). Clinical prognostic indicators of surgical outcome in cervical spondylotic myelopathy. J Neurosurg Spine.

[13] Machino M, Yukawa Y, Imagama S (2016). Surgical treatment assessment of cervical laminoplasty using quantitative performance evaluation in elderly patients: a prospective comparative study in 505 patients with cervical spondylotic myelopathy. Spine (Phila Pa 1976).

[14] Tetreault L, Palubiski LM, Kryshtalskyj M (2018). Significant predictors of outcome following surgery for the treatment of degenerative cervical myelopathy: a systematic review of the literature. Neurosurg Clin N Am.

[15] Suri A, Chabbra RP, Mehta VS, Gaikwad S, Pandey RM (2003). Effect of intramedullary signal changes on the surgical outcome of patients with cervical spondylotic myelopathy. Spine J.

[16] Tanaka J, Seki N, Tokimura F, Doi K, Inoue S (1999). Operative results of canal-expansive laminoplasty for cervical spondylotic myelopathy in elderly patients. Spine (Phila Pa 1976).

[17] Arnasson O, Carlsson CA, Pellettieri L (1987). Surgical and conservative treatment of cervical spondylotic radiculopathy and myelopathy. Acta Neurochir (Wien).

[18] Karpova A, Arun R, Davis AM (2013). Predictors of surgical outcome in cervical spondylotic myelopathy. Spine (Phila Pa 1976).

[19] Zileli M, Maheshwari S, Kale SS, Garg K, Menon SK, Parthiban J (2019). Outcome measures and variables affecting prognosis of cervical spondylotic myelopathy: WFNS Spine Committee recommendations. Neurospine.

[20] Fujiwara K, Yonenobu K, Ebara S, Yamashita K, Ono K (1989). The prognosis of surgery for cervical compression myelopathy. An analysis of the factors involved. J Bone Joint Surg Br.

[21] Wang LF, Zhang YZ, Shen Y, Su YL, Xu JX, Ding WY, Zhang YH (2010). Using the T2-weighted magnetic resonance imaging signal intensity ratio and clinical manifestations to assess the prognosis of patients with cervical ossification of the posterior longitudinal ligament. J Neurosurg Spine.

[22] Kohno K, Kumon Y, Oka Y, Matsui S, Ohue S, Sakaki S (1997). Evaluation of prognostic factors following expansive laminoplasty for cervical spinal stenotic myelopathy. Surg Neurol.

[23] Morio Y, Yamamoto K, Kuranobu K, Murata M, Tuda K (1994). Does increased signal intensity of the spinal cord on MR images due to cervical myelopathy predict prognosis?. Arch Orthop Trauma Surg.

[24] Yone K, Sakou T, Yanase M, Ijiri K (1992). Preoperative and postoperative magnetic resonance image evaluations of the spinal cord in cervical myelopathy. Spine (Phila Pa 1976).

[25] Kwok SS, Cheung JP (2020). Surgical decision-making for ossification of the posterior longitudinal ligament versus other types of degenerative cervical myelopathy: anterior versus posterior approaches. BMC Musculoskelet Disord.

[26] Shamji MF, Mohanty C, Massicotte EM, Fehlings MG (2016). The association of cervical spine alignment with neurologic recovery in a prospective cohort of patients with surgical myelopathy: analysis of a series of 124 cases. World Neurosurg.

[27] Luo J, Cao K, Huang S (2015). Comparison of anterior approach versus posterior approach for the treatment of multilevel cervical spondylotic myelopathy. Eur Spine J.

